# Prognostic Significance of EDIL3 Expression and Correlation with Mesenchymal Phenotype and Microvessel Density in Lung Adenocarcinoma

**DOI:** 10.1038/s41598-017-08851-9

**Published:** 2017-08-17

**Authors:** Dongjun Jeong, Seona Ban, Seunghyun Oh, Su Jin Lee, Seong Yong Park, Young Wha Koh

**Affiliations:** 1 Department of Pathology, College of Medicine, Soonchunhyang University, Chonan, Republic of Korea; 2 Soonchunhyang medical science research institute, College of medicine, Soonchunhyang University, Chonan, Republic of Korea; 3 Department of Nuclear Medicine and Molecular Imaging, Ajou University School of Medicine, Suwon, Republic of Korea; 4 Department of Thoracic and Cardiovascular Surgery, Yonsei University College of Medicine, Seoul, Republic of Korea; 5 Department of Pathology, Ajou University School of Medicine, Suwon, Republic of Korea

## Abstract

We examined the prognostic significance of Epidermal Growth Factor-like repeats and Discoidin I-Like Domains 3 (EDIL3) expression and its correlations with mesenchymal phenotype and microvessel density in non-small cell lung carcinoma (NSCLC). A total of 268 NSCLC specimens were evaluated retrospectively by immunohistochemical staining for EDIL3, EMT markers (e-cadherin, β-catenin, and vimentin), and CD31 to measure microvessel density. EDIL3, e-cadherin, β-catenin, and vimentin were expressed in 16%, 22.8%, 3.7%, and 10.1% of the specimens, respectively. The mRNA level of EDIL3 in tumor was correlated with the level of EDIL3 protein expression using immunohistochemistry. In lung adenocarcinoma patients, EDIL3 expression was significantly correlated with low e-cadherin expression, high vimentin expression, and increased microvessel density (*P* < 0.001, *P* = 0.001, and *P* = 0.023, respectively). In lung squamous cell carcinoma patients, EDIL3 expression was significantly correlated with low e-cadherin expression and high vimentin expression (*P* = 0.021 and *P* = 0.002, respectively). In lung adenocarcinoma patients, EDIL3 was an independent prognostic factor for overall survival in a multivariate analysis (hazard ratio: 2.552, *P* = 0.004). EDIL3 is significantly correlated with mesenchymal phenotype, angiogenesis, and tumor progression in lung adenocarcinoma.

## Introduction

Non–small cell lung cancer (NSCLC) remains one of the most deadly malignancies, with frequent metastasis and recurrence^1^. The majority of NSCLC patients are diagnosed in advanced stages. They are ineligible for potentially curative resection^1^. The 5-year survival rate of patients treated with chemotherapy for advanced NSCLC is less than 5%. They are at increased risk for side effects of chemotherapy^2^. Despite recent advances in our understanding of the genetic landscape of NSCLC, the molecular mechanisms underlying progression of NSCLC remain unclear^3, 4^. Therefore, a thorough understanding of the molecular mechanisms underlying the progression of NSCLC is urgently needed.

Epidermal Growth Factor-like repeats and Discoidin I-Like Domains 3 (EDIL3, also known as developmentally regulated endothelial cell locus 1 or DEL-1) is a secreted glycoprotein associated with endothelial cell surfaces and the extracellular matrix^5, 6^. Previous studies have documented increased expression of EDIL3 in breast cancer^7^, colon cancer^8^, bladder cancer^9^, and hepatocellular carcinoma^10^. Overexpression of EDIL3 can contribute to carcinogenesis by reducing apoptosis in cancer cells and promoting cancer vascularization^11^. Therefore, EDIL3 may be a good candidate target for developing novel cancer anti-angiogenic therapies.

The epithelial–mesenchymal transition (EMT) is a mechanism by which differentiated epithelial cells adopt a mesenchymal phenotype with loss of cell-cell junctions and loss of cellular polarity^12^. The mesenchymal phenotype is associated with enhanced migratory activity, increased resistance to apoptosis, and overproduction of extracellular matrix components^12^. E-cadherin, β-catenin, and vimentin have been used to identify cells undergoing EMT^13, 14^. Loss of e-cadherin, nuclear localization of β-catenin, and upregulation of vimentin are validated characteristics of cancer cells, highlighting the occurrence of EMT in cancer^13, 14^.

A recent study revealed that EDIL3 expression is significantly correlated with the mesenchymal phenotype in hepatocellular carcinoma^15^. In addition, overexpression of the EDIL3 gene enhances the features of EMT^15^. It promotes cancer cell proliferation and invasion in a non-small lung carcinoma cell line^16^. Therefore, it may be a specific prognostic factor in adult solid tumors. Although associations among EDIL3, mesenchymal phenotype, and angiogenesis have been observed in several malignancies^11, 15^, no study has examined the relationships among EDIL3, mesenchymal phenotype, and angiogenesis or the prognostic significance of EDIL3 expression in NSCLC patients. Therefore, the objective of this retrospective study was to evaluate the expression levels of EDIL3, the EMT markers e-cadherin, β-catenin, and vimentin, and microvessel density (MVD) using CD31 in NSCLC patients via immunohistochemical staining to determine the correlations among these markers and assess the prognostic significance of EDIL3 expression in NSCLC.

## Materials and Methods

### Patients

This retrospective study was approved by the Institutional Review Board of Ajou University School of Medicine. Informed consent was waived owing to the retrospective nature of this study. All analyses were performed in accordance with ethical guidelines for clinical research at the respective institutions. A total of 268 patients who were confirmed to have NSCLC after surgical resection between January 2009 and December 2013 were enrolled in the analysis. Clinical information including age, gender, smoking history, pathologic tumor/node/metastasis (TNM) stage, adjuvant chemotherapy, and adjuvant radiotherapy were obtained from medical records. The median follow-up time was 38.2 months (range: 1–85.9 months).

### Histopathological analysis and immunohistochemistry

Histological subclassification was performed by one pathologist (YWK) according to the 2015 World Health Organization Classification of Lung Tumors^17^. Representative tumor section paraffin blocks were arrayed with a tissue-arraying instrument. Each sample was arrayed in two 2 mm diameter cores to minimize tissue loss and overcome tumor heterogeneity. Tissue microarray sections were arranged in a Benchmark XT automatic immunohistochemical staining device (Ventana Medical Systems, Tucson, AZ, USA). Samples were incubated with antibodies against EDIL3 (polyclonal, ab198003, Abcam), e-cadherin (monoclonal, 36B5, Novocastra), vimentin (monoclonal, V9, Novocastra), β-catenin (monoclonal, β-catenin1, DAKO), and CD31 (monoclonal, JC70, Cell Marque).

**Figure 1:**
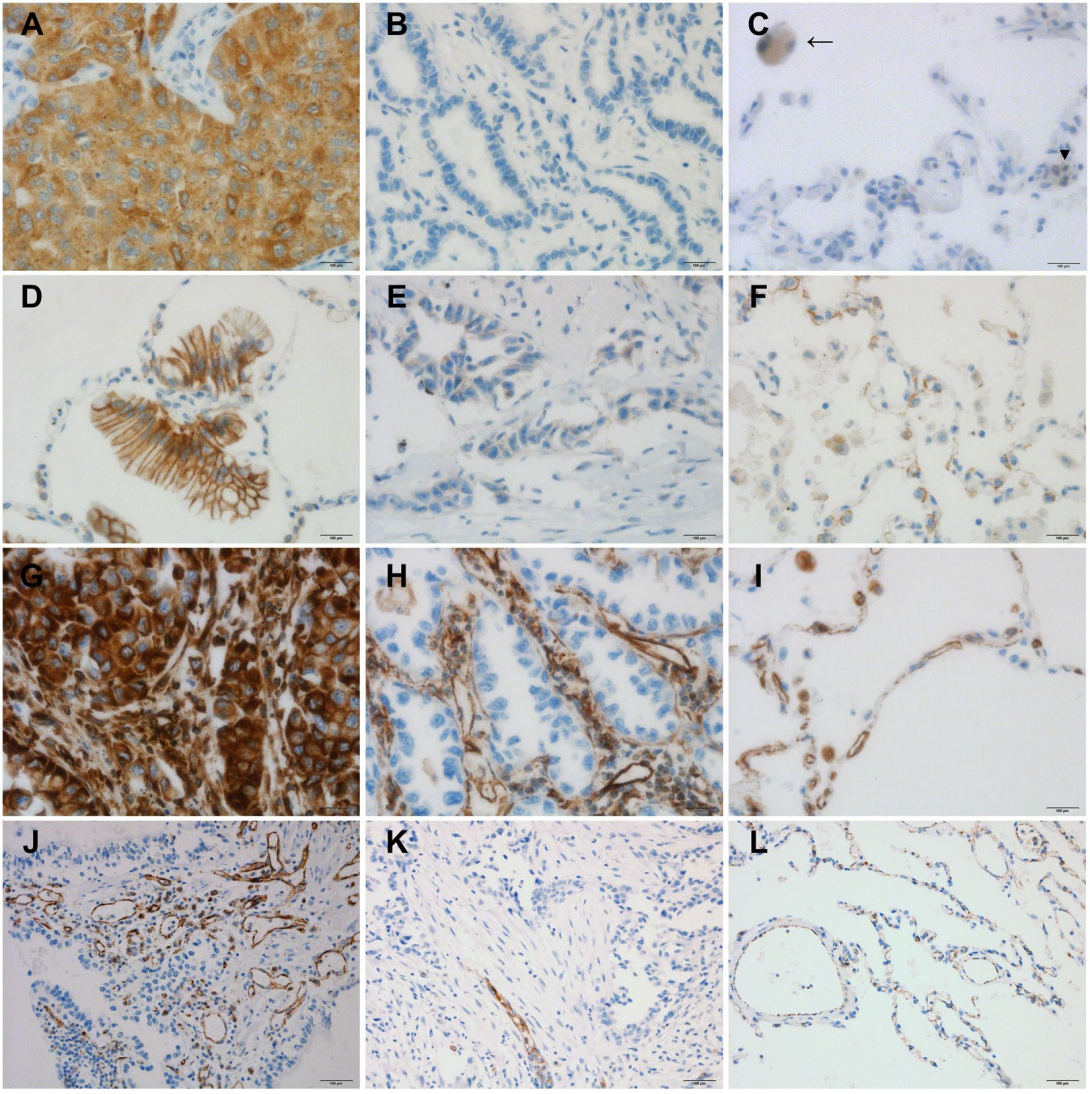
EDIL3, e-cadherin, vimentin, and CD31 expression in lung adenocarcinoma and tumor-free control lung samples. (**A**) Positive EDIL3 expression on tumor cells(x400). (**B**) Negative EDIL3 expression on tumor cells(x400). (C) Faint positive EDIL3 expression on macrophages (arrow) and lymphocyte (arrowhead) and negative expression of pneumocyte and endothelial cells of control sample (x400). (**D**) Positive e-cadherin expression on tumor cells(x400). (**E**) Negative e-cadherin expression on tumor cells(x400). (**F**) Positive e-cadherin expression on pneumocyte of control sample(x400). (**G**) Positive vimentin expression on tumor cells(x400). (**H**) Negative vimentin expression on tumor cells(x400). (**I**) Positive vimentin expression on capillary and macrophage of control sample (x400). (**J**) High microvessel density with CD31 expression on tumor. (x200). (**K**) Low microvessel density with CD31 expression on tumor(x200). (**L**) Positive CD31 expression on capillary of control sample (x200).

We performed EDIL3 immunohistochemistry in 8 tumor-free control lung samples. Tumor-free control lung samples were taken from patients who underwent surgical resection for subpleural bullae. Macrophages and lymphocytes showed a faint positive response to EDIL3 in tumor-free control lung samples, however pneumocytes, bronchial epithelium and endothelial cells were negative for EDIL3 immunohistochemistry. We counted tumor cells to compare the expression levels of EDIL3, e-cadherin, β-catenin, and vimentin in the lung sections. Tumor-specific immunohistochemistry (napsinA for adenocarcinoma and p40 for squamous cell carcinoma) was performed prior to the experiment to determine the location of tumor cells. For each case, all tumor cells were analyzed and the percentage of positively stained tumor cells was recorded (positively stained tumor cells/tumor cells). The staining results were scored based on staining intensity: 0 (no staining), 1 (faint staining = light yellow), 2 (moderate staining = yellow-brown), and 3 (strong staining = brown) (Fig. 1A–C). Cases with a score of 2 or 3 were considered positive. The percentage of tumor cells expressing EDIL3 that showed the most significant difference with respect to overall survival (OS) was selected as the cutoff value for defining the high- and low-Edil3 groups. That cutoff value was 10%. This immunohistochemical scoring system has been used in previous studies^18–20^. For the EMT markers, a four-tier scoring system (0, 1, 2, and 3) was used, and cases with scores of 2 or 3 were considered high. Previous studies have set the epithelial–mesenchymal transition biomarker cutoff as 50 or 60%^21–23^. Therefore, we set the epithelial–mesenchymal transition biomarker cutoff as 50%. For E-cadherin, two categories were established: low (<50% of membranous stained cells) and high (>50% of membranous stained cells) (Fig. 1D–F). For β-catenin, two categories were established: nuclear expression (>50% of nuclear stained cells) and membranous expression (>50% of membranous stained cells). For vimentin, a 50% cutoff was used to define the staining pattern. When at least 50% of tumor cells exhibited vimentin cytoplasmic staining, the tumor was considered as vimentin high (Fig. 1G–I). We also examined the number of MVD, as previously described^24^. To count microvessels, the area with the highest vascularization was selected at low magnification (x100). Counting was performed at high magnification (x400) (Fig. 1J–L). Three fields were examined per case. The final MVD for each case was presented as the mean value of the three fields examined. Microvessels with a clearly defined lumen or a well-defined linear vessel shape were selected for counting. Branching vessel structures were considered as a single vessel.

### RNA extraction and realtime qPCR

Paraffin blocks were sectioned in 10 um and were transferred onto slides. The slides were incubated in 60 °C for 1 h followed by deparaffinization in xylene. The slides were washed in 100% ethanol and hydrated in serial ethanol in DEPC water (95%, 80% and 70%). The slides were dried. For the microdissection of the tumor and normal areas, the slides were soaked in DEPC water and microdissected with fine needle under the light microscope. The microdissected tissues were incubated in lysis buffer in MiRCURY RNA Isolation Kit (Cat. No. 300115. Venlo, Netherland) and total RNA was extracted as the manufacturer’s instructions. The cDNA was synthesized by Toyobo ReverTra Ace qPCR RT Kit (Cat. No. FSQ-101. Osaka, Japan) with random examer, followed by real-time PCR with Toyobo SYBR Green Real-time PCR Master Mix (Cat. No. FSQ-101) as manufacturer’s instructions. The primer sequences and amplified nucleotide length were: GAPDH (140 bp); forward, 5′-ACGACCACTTTGTCAAGCTC-3′, reverse, 5′-TCTTCCTCTTGTGCTCTT GC-3′. EDIL3 (112 bp); forward, 5′-GCGAATGGAACTTCTTGGCTGTG-3′; reverse, 5′-GAGC GTTCTGAAGATGCTGGAG-3′. The real-time qPCR was run in BioRad CFX Connect System (California, USA). The PCR program consisted of 95 °C for 10 sec, 57 °C for 10 sec and 72 °C for 30 sec. and run by 40 cycles. The relative expression of mRNA was calculated by 2ˆ(−delta delta CT) method^25^. In each run, the templates were assayed in triplicate and the run was repeated twice.

### Statistical analyses

OS was defined as the time between the day of diagnosis and the day of death regardless of the cause of death. The follow-up for patients still alive was censored at their last follow-up date. OS was analyzed with a Kaplan–Meier curve. Values were compared with the log-rank test. Multivariate prognostic analysis of OS was performed using a Cox proportional hazards regression model. Predictors with a *p* value of ≤0.05 in univariate analysis together with clinically important variables were included in the final multivariate analysis. The enter method was employed to determine the final Cox model for multivariate analysis. Categorical variables were compared using the chi-squared test, while continuous variables were compared using the independent-sample *t*-test. All statistical analyses were performed using the SPSS statistical software (version 18.0; SPSS; Chicago, IL, USA). Statistical significance was considered at p < 0.05.

## Results

### Patient demographics

The demographic characteristics of the patients included in this study are summarized in Table 1. Patient age ranged from 35 to 86 years (median, 64 years). The number of patients with pathologic TNM stage I, II, and III was 132 (51%), 69 (26.6%), and 58 (21.6%), respectively. The number of patients with adenocarcinoma and squamous cell carcinoma was 166 (61.9%) and 102 (38.1%), respectively. Most patients (67.9%) had a history of smoking.

**Table 1:** Demographic and clinical characteristics of the study subjects.

Variable	Number (%)
Age, median (range), years	64 (35–86)
Male sex	190 (70.9%)
Smoking history	171 (67.9%)
Operation	
Pneumonectomy	15 (5.6%)
Lobectomy	218 (81.3%)
Wedge resection	35 (13.1%)
pT stage	
T1/T2	47 (17.5%)/202 (75.4%)
T3/T4	16 (6%)/3 (1.1%)
pN stage	
NX/N0/N1	9 (3.4%)/157 (58.6%)/47 (17.5%)
N2/N3	53 (19.8%)/2 (0.7%)
pTNM 7th edition	
Stage I	132 (51%)
Stage II	69 (26.6%)
Stage III	58 (21.6%)
Histologic subtype	
Adenocarcinoma	166 (61.9%)
Squamous cell carcinoma	102 (38.1%)
Adjuvant chemotherapy	76 (28.5%)
Adjuvant EGFR TKI	16 (9.2%)
Adjuvant radiotherapy	92 (34.5%)

EGFR TKI: epidermal growth factor receptor tyrosine kinase inhibitor.

### EDIL3, EMT markers, and microvessel density in NSCLC tissues

Forty-four (16%) patients showed cytoplasmic and membranous positivity for EDIL3. The number of EDIL3-positive patients with adenocarcinoma and squamous cell carcinoma was 35/166 (21.1%) and 9/102 (8.8%), respectively. Sixty-one (22.8%) patients showed reduced membrane expression of e-cadherin. Ten (3.7%) patients showed nuclear expression of β-catenin. Twenty-seven (10.1%) patients showed cytoplasmic expression of vimentin. The mean MVD of all cases was 10.99 ± 5.18 (standard deviation (SD)) (range, 3.3–26.6).

### Correlation between EDIL3 protein expression and EDIL3 mRNA expression

**Figure 2:**
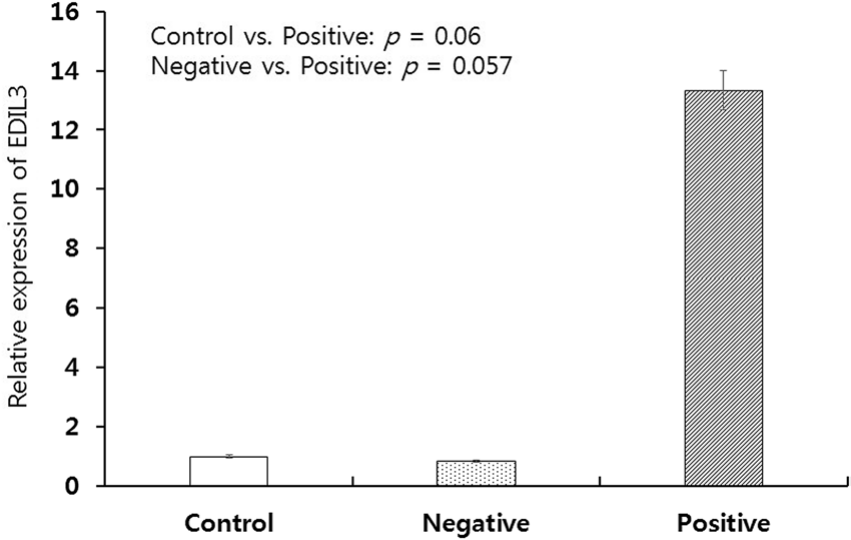
Correlation between EDIL3 protein expression and EDIL3 mRNA expression. The mRNA level in tumors positive for EDIL3 immunohistochemistry was higher than that in tumor-free control lung samples and tumors without EDIL3 immunohistochemical positivity.

To support the results of protein expression by immunohistochemistry in normal and cancer tissues, we performed real-time qRT PCR in normal, EDIL3 negative cancer and EDIL3 positive cancer respectively. A total of 24 paraffin blocks consisted of 8 of normal, 8 of EDIL3 low expression cancer and 8 of EDIL3 high expression were evaluated. The mRNA level in tumors positive for EDIL3 immunohistochemistry was higher than that in tumor-free control lung samples (*p* = 0.06, Fig. 2) and tumors without EDIL3 immunohistochemical positivity (*p* = 0.057, Fig. 2).

### Correlation among EDIL3, EMT markers, and microvessel density

**Figure 3:**
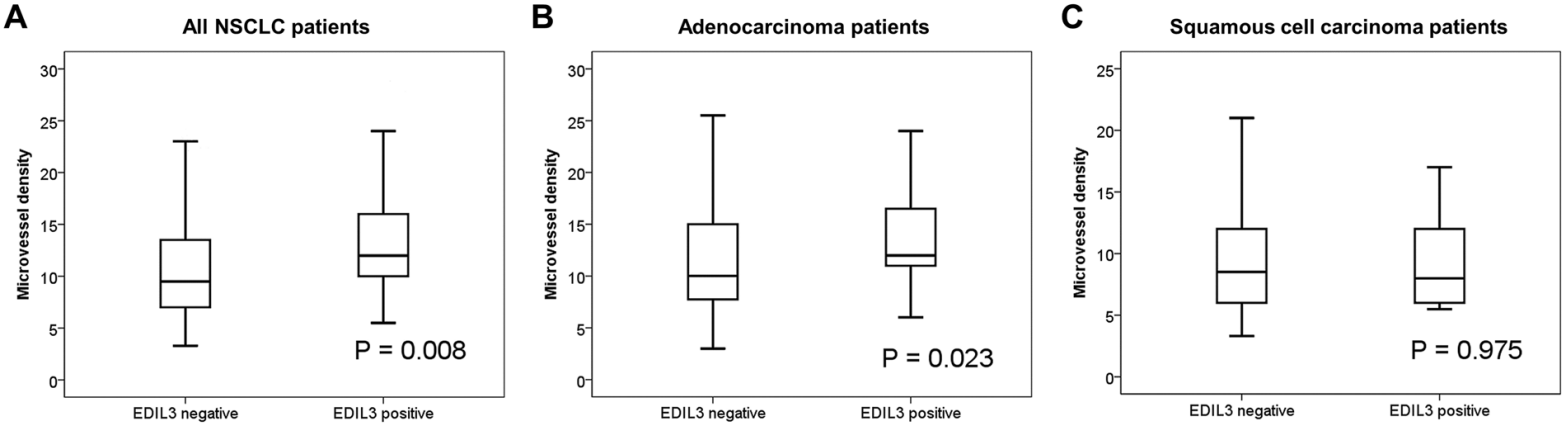
Correlation between EDIL3 and microvessel density. (**A**) All NSCLC patients. (**B**) adenocarcinoma patients. (**C**) squamous cell carcinoma patients.

In the EDIL3-positive group, there was lower e-cadherin expression than in the negative group in all cases (*p* < 0.001, Table 2). Vimentin expression was higher in the EDIL3-positive group than in the EDIL3-negative group (*p* < 0.001, Table 2). However, there was no correlation between EDIL3 expression and nuclear β-catenin expression (*p* = 0.215, Table 2). The mean microvessel density in tumors positive for EDIL3 was significantly (*p* = 0.008) higher than that in tumors negative for EDIL3 in all cases (Fig. 3A).

**Table 2:** Correlation of EDIL-3 expression with e-cadherin, vimentin, and β-catenin expression.

Characteristic	EDIL3 expression in all cases		*p*	EDIL3 expression in adenocarcinoma cases		*p*	EDIL3 expression in squamous cell carcinoma cases		*p*
	Negative (*n* = 224)	Positive (*n* = 44)		Negative (*n* = 131)	Positive (*n* = 35)		Negative (*n* = 93)	Positive (*n* = 9)	
E-cadherin expression			<0.001^†^			<0.001^†^			0.021^‡^
Reduced	38 (17%)	23 (52.3%)		21 (16%)	18 (51.4%)		17 (18.3%)	5 (55.6%)	
Preserved	186 (83%)	21 (47.7%)		110 (84%)	17 (48.6%)		76 (81.7%)	4 (44.4%)	
Vimentin expression			<0.001^‡^			0.001^‡^			0.002^‡^
Negative	211 (94.2%)	30 (68.2%)		122 (93.1%)	25 (71.4%)		89 (95.7%)	5 (55.6%)	
Positive	13 (5.8%)	14 (31.8%)		9 (6.9%)	10 (29.6%)		4 (4.3%)	4 (44.4%)	
β-catenin expression			0.215^†^			0.639^†^			0.244^†^
Negative	217 (96.9%)	41 (93.2%)		126 (96.2%)	33 (94.3%)		91 (97.8%)	8 (88.9%)	
Positive	7 (3.1%)	3 (6.8%)		5 (3.8%)	2 (5.7%)		2 (2.2%)	1 (11.1%)	

^†^Chi-squared test by two-sided Pearson’s exact test; ^‡^Chi-squared test by two-sided Fisher’s exact test.

We then performed subgroup analysis according to histologic subtypes. In adenocarcinoma patients, EDIL3 expression was significantly correlated with low e-cadherin expression (*P* < 0.001), high vimentin expression (*P* = 0.001), and increased microvessel density (*P* = 0.023) (Table 2, Fig. 3B). However, there was no correlation between EDIL3 expression and nuclear β-catenin expression in adenocarcinoma patients (*P* = 0.639, Table 2). In squamous cell carcinoma patients, EDIL3 expression was significantly correlated with low e-cadherin expression (*P* = 0.021) and high vimentin expression (*P* = 0.002) (Table 2). However, EDIL3 expression was not significantly associated with nuclear β-catenin expression (*P* = 0.244) or microvessel density (*P* = 0.975) (Table 2, Fig. 3C).

### Prognostic significance of EDIL3 expression

**Figure 4:**
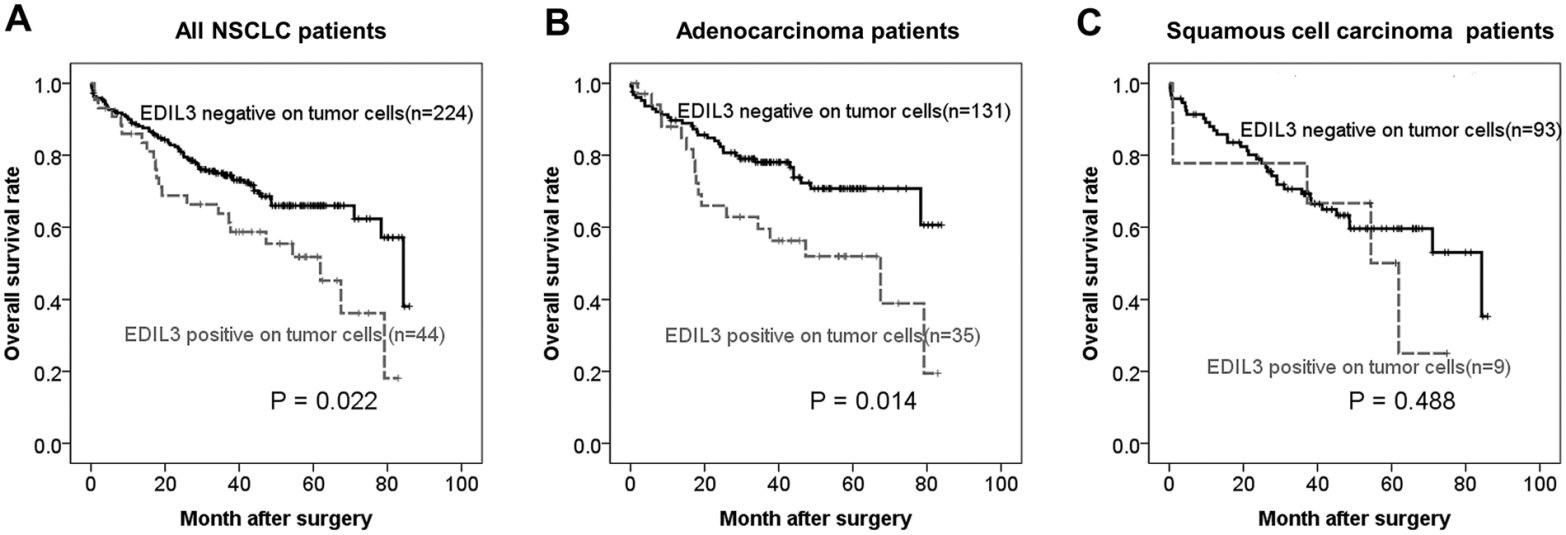
Comparison of survival rates according to EDIL3 expression in tumor cells. (**A**) All NSCLC patients. (**B**) adenocarcinoma patients. (**C**) squamous cell carcinoma patients.

EDIL3-positive patients had lower 5-year OS rates than did EDIL3-negative patients in all cases (51.7% vs. 66.0%, *P* = 0.022; Fig. 4A). Among adenocarcinoma patients, EDIL3-positive patients had lower 5-year OS rates than did EDIL3-negative patients (51.9% vs. 70.7%, *P* = 0.014; Fig. 4B). However, in squamous cell carcinoma patients, EDIL3 positivity was not significantly (*P* = 0.488) associated with OS (Fig. 4C).

In the univariate analysis, OS was significantly (*P* < 0.05) associated with age (≥65 years), male gender, and pathologic stage in adenocarcinoma patients. In the multivariate analysis, EDIL3 expression was an independent prognostic marker of OS in adenocarcinoma patients (hazard ratio: 2.552, *P* = 0.004; Table 3). Patients with EDIL3 expression were more likely to have the mucinous adenocarcinoma subtype (17.6% vs. 2.3%) and less likely to have the solid adenocarcinoma subtype (11.8% vs. 22.1%) than were EDIL3-negative patients (*P* = 0.004; Table 4). However, EDIL3 was not significantly (*P* > 0.05) associated with lymphovascular invasion, pathologic TNM stage, or smoking history in adenocarcinoma patients (Table 4).

**Table 3:** Multivariate analysis of overall survival in lung adenocarcinoma patients.

Variables	HR	95% CI	*p*
Age (<65 vs. ≥65)	1.532	0.844–2.782	0.161
Sex (female vs. male)	3.153	1.537–6.489	0.002
Pathologic stage			
I		reference	
II	1.773	0.748–4.205	0.194
III	3.340	1.699–6.568	<0.001
EDIL-3 (− vs.+)	2.552	1.341–4.858	0.004

**Table 4:** Correlation of EDIL-3, e-cadherin, and vimentin expression and pathologic characteristics in patients with adenocarcinoma.

Characteristic	EDIL-3 expression		*p*	E-cadherin expression		*p*	Vimentin expression		*p*
	Negative (*n* = 131)	Positive (*n* = 34)		Reduced (*n* = 30)	Preserved (*n* = 135)		Negative (*n* = 147)	Positive (*n* = 18)	
Predominant histologic subtype			<0.028^†^			0.249^†^			0.285^†^
Acinar	65 (49.6%)	14 (41.2%)		13 (43.3%)	66 (48.9%)		72 (49%)	7 (38.9%)	
Lepidic	9 (6.9%)	2 (5.9%)		0 (0%)	11 (8.1%)		11 (7.5%)	0 (0%)	
Papillary	20 (15.3%)	7 (20.6%)		7 (23.3%)	20 (14.8%)		23 (15.6%)	4 (22.2%)	
Micropapillary	5 (3.8%)	1 (2.9%)		0 (0%)	6 (4.4%)		6 (4.1%)	0 (0%)	
Solid	29 (22.1%)	4 (11.8%)		9 (30%)	24 (17.8%)		26 (17.7%)	7 (38.9%)	
Mucinous	3 (2.3%)	6 (17.6%)		1 (3.3%)	8 (5.9%)		9 (6.1%)	0 (0%)	
Positive lymphovascular invasion	52 (39.7%)	15 (42.9%)	0.847^‡^	11 (36.7%)	56 (41.2%)	0.686^‡^	59 (40.1%)	8 (42.1%)	>0.99^‡^
pTNM 7th edition			0.912ª			0.150ª			0.890ª
Stage I	68 (53.5%)	18 (54.5%)		14 (46.7%)	72 (55.4%)		75 (52.8%)	11 (61.1%)	
Stage II	22 (17.3%)	6 (18.2%)		9 (30%)	19 (14.6%)		25 (17.6%)	3 (16.7%)	
Stage III	37 (29.1%)	9 (27.3%)		7 (23.3%)	39 (30%)		42 (29.6%)	4 (22.2%)	
Positive smoking history	64 (54.2%)	17 (51.5%)	0.845^‡^	14 (58.3%)	67 (52.8%)	0.661^†^	70 (51.9%)	11 (68.8%)	0.290^‡^

^†^Chi-squared test by two-sided Fisher’s exact test; ^‡^Chi-squared test by two-sided Pearson’s exact test.

ªChi-squared test by two-sided linear-by-linear association.

## Discussion

Our study provides several novel findings. First, it shows the correlation between EDIL3 expression and mesenchymal phenotype, characterized by low e-cadherin and enhanced vimentin expression in NSCLC patients. Secondly, the mRNA level of EDIL3 in tumor was correlated with the level of EDIL3 protein expression using immunohistochemistry. Thirdly, this study revealed that EDIL3 expression is significantly associated with tumor angiogenesis, characterized by microvessel density in lung adenocarcinoma tissue. Fourthly, our study demonstrates that EDIL3 expression has prognostic value in lung adenocarcinoma. These results suggest that EDIL3 expression may promote tumor progression through enhancing EMT and tumor angiogenesis in lung adenocarcinoma.

EDIL3 is associated with tumor angiogenesis and EMT. It plays a critical role in the interaction between hepatocellular carcinoma and endothelial cells^11, 26^. Furthermore, murine Lewis lung carcinoma cells engineered to express EDIL3 have a 2- to 4-fold increase in capillary density and an accelerated growth rate^11^. A previous study confirmed that overexpression of the EDIL3 gene can enhance features of EMT, increasing vimentin while decreasing E-cadherin in a lung cancer cell line^16^. Xia *et al*. also demonstrated that EDIL3 expression is higher in hepatocellular carcinoma cells with a mesenchymal phenotype than in those with an epithelial phenotype^15^. In the present study, we found that EDIL3 expression is closely associated with mesenchymal phenotype. EMT induces tumor angiogenesis. This is the most important step in the transition of tumors from a primary state to a malignant one^27, 28^. Overexpression of angiopoietin 2 can promote EMT-induced angiogenesis in oral squamous cell carcinoma^29^. It has been shown that EMT can confer efficient tumorigenicity by enhancing the expression of the proangiogenic factor VEGF-A and by increasing tumor angiogenesis in a murine breast cancer model^30^.

Previous mRNA blot analysis reported that EDIL3 expression was found to be restricted to endothelial cells in the embryo but not the adult in mice^31^. However, other real-time reverse transcription polymerase chain reaction (RT-PCR) study demonstrated that EDIL3 is expressed in brain and lung, but has little or no expression in the liver, kidney, spleen, or heart in mice adults cells^32^. A web-based database enabling exploration of individual proteins reported that EDIL3 expression is present only in macrophages of normal human lung samples and not in pneumocytes^33^. Our results also identified the protein expression of EDIL3 on some macrophages and lymphocyte of tumor-free control samples however pneumocytes, bronchial epithelium and endothelial cells were negative for EDIL3 immunohistochemistry.

In the present study, EDIL3 expression was found to be correlated with microvessel density and poor outcome in adenocarcinoma cases. However, there was no association between EDIL3 expression, microvessel density, or clinical outcome in squamous cell carcinoma. The percentage of EDIL3 positivity was also significantly higher in adenocarcinoma than in squamous cell carcinoma (21% vs. 8%, *p* = 0.01). Adenocarcinoma is more closely associated with EMT and angiogenesis than is squamous cell carcinoma. Kim *et al*. revealed that expression of the mesenchymal marker vimentin is significantly higher in adenocarcinoma than in squamous cell carcinoma^34^. Vascular density has previously been found to be significantly higher in adenocarcinomas than in squamous cell carcinomas^35^. These results suggest that adenocarcinoma may include more EDIL3-positive cells and respond more robustly to EDIL3 than squamous cell carcinoma, resulting in tumor angiogenesis, presence of a mesenchymal phenotype, and poor clinical outcome.

In this study, groups that expressed EDIL3 were more likely to have the mucinous adenocarcinoma subtype than were EDIL3-negative groups. Mucinous adenocarcinoma is strongly correlated with KRAS (Kirsten rat sarcoma viral oncogene homolog) mutations^36^. Nadal *et al*. reported that KRAS-G12C mutants overexpress EMT genes in surgically resected lung adenocarcinoma^37^. Activation of KRAS signaling could stimulate EMT pathways via extracellular signal–regulated kinase (ERK)1/2 in lung cancer cells^38^. MiR-134 inhibited cell proliferation and EMT by targeting KRAS in a renal cell carcinoma cell line^39^.

Several attempts have been made to explore the therapeutic effect of EDIL3 in cancer. Downregulation of EDIL3 with small interfering RNA gene therapy has been shown to suppress the growth of colon tumors by inhibiting angiogenesis and cell proliferation in a mouse model^8^. Xia *et al*. reported that EDIL3 overexpression can activate the TGF-β and ERK signaling pathway by interacting with αvβ3 integrin^15^. Blocking the TGF-beta and ERK signaling pathway can effectively reduce EDIL3-mediated angiogenesis and invasion in a hepatocellular carcinoma mouse model^15^. It has been reported that knockdown of EDIL3 by shRNA-containing plasmids promotes anoikis and inhibits anchorage-independent tumor growth in a pancreatic ductal adenocarcinoma cell line^40^.

The retrospective design is one limitation of this study. The tissue microarray design could not reflect a whole tumor section because of the heterogeneous distribution of immunohistochemical staining. The number of patients with squamous cell carcinoma was relatively small and the incidence of EDIL3+ or Vimentin+ was low. A further large-scale study is needed to evaluate the roles of EDIL3 and vimentin in squamous cell carcinoma.

In conclusion, EDIL3 overexpression is an independent negative prognostic factor for OS in lung adenocarcinoma. EDIL3 overexpression is correlated with mesenchymal phenotype and increased microvessel density. The EDIL3/EMT/angiogenesis pathway may provide further insight into tumor progression in lung adenocarcinoma. It may be useful as a novel target of therapeutic modalities for lung adenocarcinoma.
